# Systemic candidiasis in farm-reared red-legged partridges (*Alectoris rufa*) caused by *Leucosporidium* spp

**DOI:** 10.1186/1746-6148-8-81

**Published:** 2012-06-18

**Authors:** Giovanni Lanteri, Francesco Macrì, Giuseppe Rapisarda, Florinda Basile, Stefano Reale, Fabio Marino

**Affiliations:** 1 Dip. Sanità Pubblica Veterinaria, Università degli Studi di Messina, Polo Universitario dell’Annunziata, 98168, Messina, Italy; 2 Medico Veterinario, Collaboratore Esterno, Messina, Italy; 3 Istituto Zooprofilattico Sperimentale della Sicilia “A. Mirri”, Via Gino Marinuzzi 3, 90129, Palermo, Italy

**Keywords:** *Alectoris rufa*, Candidiasis, *Leucosporidium scottii*, Pathology, PCR, Radiology, Red legged partridge

## Abstract

**Background:**

This report describes the results of radiological, histological and molecular examination of three farm-reared red-legged partridges (*Alectoris rufa*) affected by candidiasis.

**Case presentation:**

Three juvenile farm-reared red-legged partridges in a batch of 100 of the same species were sent for clinical and pathological investigations. The owner referred of a sudden isolation of the sick animals, with apathy, diarrhea, ruffled plumage and respiratory rattles. Post mortem total body lateral projection radiograph showed an increased perihilar interstitial pattern and air bronchogram signs due to lung edema. At necropsy, carcasses showed cachexia; the pericloacal region was soiled by diarrheic fecal material. From the mouth to the intestine, a mucous yellowish fluid was present on a slightly reddish mucosa. Histopathology showed slight edema and congestion with different free fungal elements, referable to blastospores, hyphae and pseudohyphae. Biomolecular exam identified the most similar sequences as belonging to *Leucosporidium scottii*.

**Conclusion:**

To our knowledge, this case report describes for the first time this fungal species as a causative agent of candidiasis in birds.

## Background

Aspergillosis, zygomycosis and candidiasis are the most frequently detected mycoses in avian species. Among these, candidiasis is a fungal disorder generally due to different species belonging to the genus *Candida*. Many of the fungal species included in this genus are today considered the most common pathogenic fungi [1], detectable in terrestrial soil and in fresh and marine water [2,3], capable of infecting human beings and animals. In poultry *C. ravautii, C. salmonicola, C. guilliermondi, C. parapsilosis, C. catenulata* and *C. brumptii* have been identified [4], and *C. albicans* alone represents about 95% of the fungal species isolated from crop. This yeast, often considered commensal, may cause disease in some avian species, such as Californian turkeys [5], captive birds [6], Japanese nightingales [7], Amazon parakeets [8], hihi [9] and red-legged partridges [10,11]. In the present paper, clinical, pathological and biomolecular features characterizing candidiasis in red-legged partridges are reported.

## Case presentation

Three 3-months-old male red-legged partridges (*Alectoris rufa*, Linnaeus 1758), suddenly died in October 2010, were sent to the Department of Veterinary Public Health, Unit of Pathology, for clinical and pathological investigations. The birds were part of a group of 100 animals belonging to the same species reared in a private farm located on a hill in the district of Messina. No other animal species were present in the farm. The owner bought the young birds from a private farm located in the northern Italy and grew them up without producing eggs by himself. The farm extended on a half hectare of land, exclusively deep litter system type. The owner built a circular box, about 10 m in diameter, enclosed with a metal net, about 2 m in height laterally and 3.5 m centrally, with no natural and artificial lairs; within this aviary, the owner put in a bedding of mixed straw and wood shaving directly on soil and rarely replaced it, feed box with low edge and iron poultry drinkers; subjects were introduced and subdivided in different classes of age. For three months-aged animals, food mainly consisted of commercial mixed pellet based on cereals, leguminous and minerals without any antimicrobials and coccidiostats. No vaccination protocols were applied. Anamnestic data collected from the owner referred of a sudden isolation of the diseased animals, with apathy, diarrhea and ruffled plumage; moreover, close to a diseased bird, respiratory rattles were be heard. When the first three birds died, early during the outbreak, suspecting zoonosis, the owner required necropsy. The farmer administered by himself the remaining partridges antibiotic therapy (enrofloxacin); over a three weeks’ period all the remaining birds died. Post mortem radiographic exam was performed prior to necropsy on the three dead subjects. Written informed consent was obtained from the owner for publication of this report and any accompanying images. Necropsy was performed following the method proposed by Taccini *et al*. [12]. Cytological imprinting smears, obtained from lungs, liver and kidneys were stained with May Grunwald Giemsa (Bio-Optica SpA, Milano, 20134, Italy). Tissue samples obtained from the following organs, lungs, liver, esophagus, crop, proventriculus, gizzard, spleen, intestine, brain and skin were collected and fixed in 10% buffered formalin and embedded in paraffin wax. Microbiological exams for anaerobic and aerobic bacteria, virus isolation for paramyxovirus and other viral agents [13], as well as parasitological examinations for intestinal protozoa and nematodes were performed to rule out other primary diseases. For the histopathological exam, 5 μm thick histological sections were obtained and stained with Haematoxylin and Eosin (Carlo Erba Reagenti Spa, Rodano, 20090, MI, Italy), as well as with two other histochemical tests helpful to identify fungal elements, Periodic Acid Schiff (PAS) (Bio-Optica SpA, Milano, 20134, Italy) and Grocott (Bio-Optica SpA, Milano, 20134, Italy). On the basis of microscopic examination of paraffin embedded tissues, molecular tests for fungal pathogens were performed by a ribosomal internal transcribed spacer targeted amplification reaction.

For this purpose the samples were pre-treated with mineral oil at 95 °C for 5 min for three times [14] to eliminate the paraffin embedding tissues. This pre-treatment is necessary to avoid PCR inhibition and permits the amplification of target DNA sequences as long as 611 nts.

Successively total DNA was extracted from lung and gizzard paraffin embedded tissue sampled from three birds by using a QIAmp DNA mini kit (QIAGEN, Hilden, 40724, Germany). The contaminations were escluded during the DNA extraction, amplification or elution steps by application of the good laboratory practice. Moreover the extraction took place in a controlled area, in sterile mode, by using disposable and filtered equipments. The DNA was specthrophotometrically quantized and employed in PCR test targeted to the ribosomal regions. The DNA sequences, known as ITS, were amplified by primers annealing at the end of 18 S and 5.8 S ribosomal genes. This approach permits the use a couple of fungal universal primers to amplify a species specific DNA sequence. Species was after revealed by application of sequencing protocol on the PCR product. Amplification of the ITS1 and ITS2 regions was performed with universal fungal primers ITS1 (5′-TCCGTAGGTGAACCTGCGG-3′) and ITS4 (5′-TCCTCCGCTTATTGATATG-3′) [15]. The full ITS region was amplified by PCR in a final reaction volume of 50 μl. Each reaction mixture contained approximately 10 ng of template DNA; 0.4 pmol (each) forward (ITS1) and reverse (ITS4) primers; 10 μM (each) dATP, dCTP, dGTP, and dTTP; 10X reaction buffer containing 1.5 mM MgCl_2_ (AB) and 2 U of Taq Gold (AB). The amplification was performed in a 9700 thermal cycler (Applied Biosystems Inc., Foster City, California, 94404, USA). An initial denaturation step (94 °C for 5 min) was followed by 35 cycles (with each cycle consisting of DNA denaturation at 94 °C for 30 s, primer annealing at 55 °C for 30 s, and elongation at 72 °C for 1 min) and a final extension step at 72 °C for 7 min. A no-template negative control was included in each PCR run. The PCR products were purified with a Spin PCR purification kit (QIAGEN, Hilden, 40724, Germany), following of the manufacturer’s protocol. The DNA was eluted in 25 μl of double-distilled H_2_O. The purified PCR products were sequenced with the primers ITS1 and ITS4 in two different reactions using BigDye Terminator cycle sequencing 1.1 kit (Applied Biosystems Inc., Foster City, California, 94404, USA). Sequencing was performed on an ABI Prism 310 DNA sequencer (Applied Biosystems Inc., Foster City, California, 94404, USA). The obtained data were analised by Wu Blast 2 sequence allignment software, considering 97% identitiy as the stringent parameter for strain identification.

**Figure 1:**
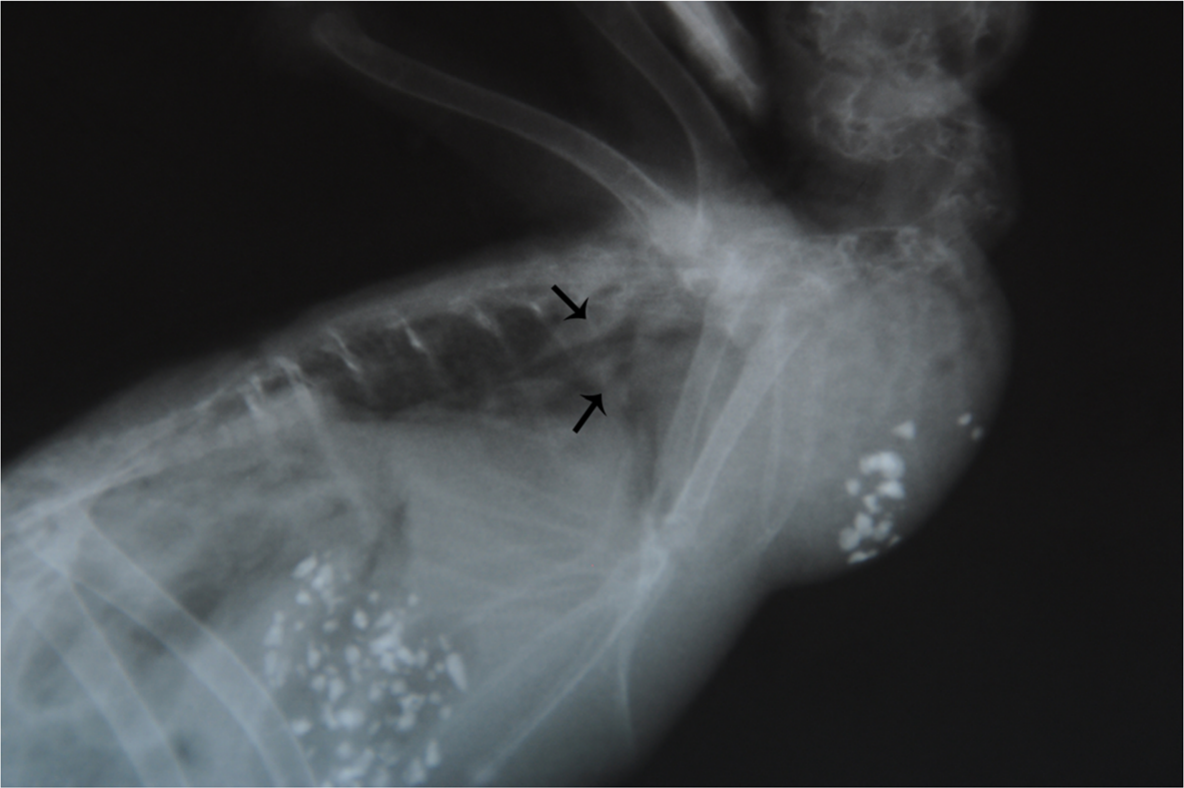
***Alectoris rufa*: X-ray.** Radiograph perihilar interstitial pattern and air bronchogram showing pulmonary oedema. Arrows.

**Figure 2:**
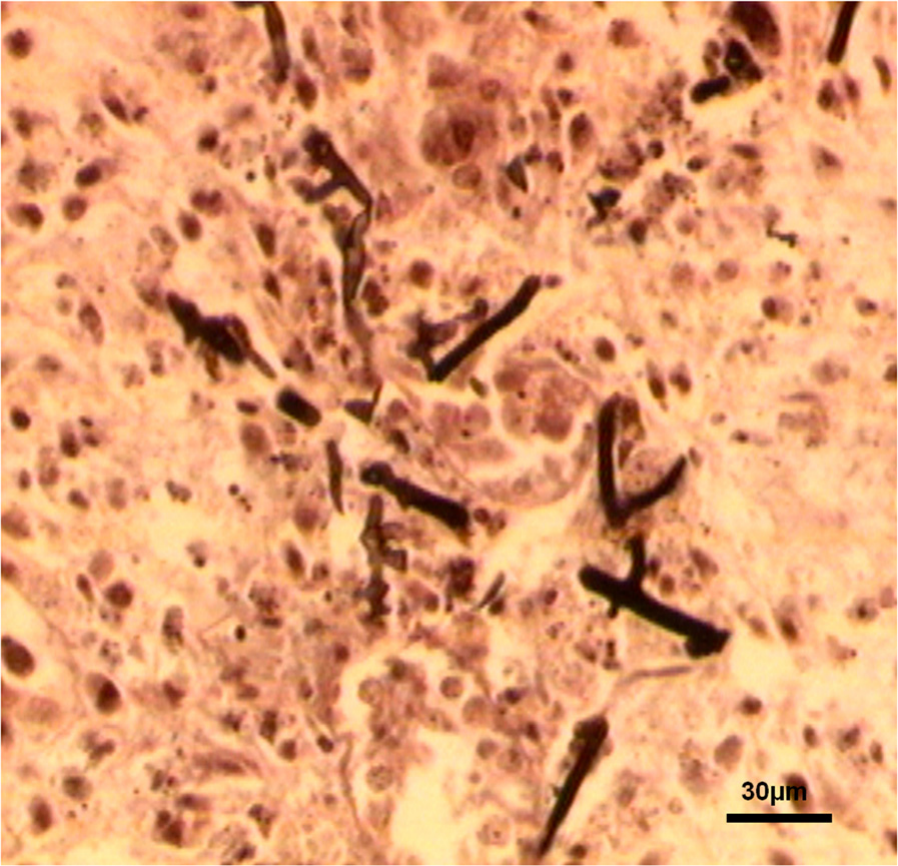
***Alectoris rufa*:*Leucosporidium scottii.*** Different fungal elements free in pulmonary tissue. (Grocott) (Bar = 30 μm).

Total body lateral projection radiograph showed an increased perihilar interstitial pattern and air bronchogram signs due to lung edema (Figure 1). At necropsy carcasses showed similar features. Birds were cachectic (70/80gr ± in weight); the pericloacal region was soiled by diarrheic fecal material. Opening the mouth, a little amount of mucous fluid finely distributed on a slightly reddish mucosa was seen; opening the abdominal cavity, similar features were detected on the esophagus, crop, proventriculus and gizzard. The intestine was dilated and filled by a yellow fluid content. Liver, lungs, kidneys and heart showed slight increase in volume when compared to normal size and were dark red in colour, suggesting a congestion; a serum haematic fluid oozed out on cut section. At microscope, cytological smears revealed several hyphae, pseudohyphae and blastospores. No further pathogens were detected or isolated. Histopathological exam of the lung, showed slight edema and congestion of the blood vessels, with different fungal elements, free in the tissue and within vessels, referable to blastospores, hyphae and pseudohyphae; similar bodies were detected in all organs and tissues; moreover very few macrophages were identified. In the esophagus, crop, proventriculus and gizzard, numerous vesicles surrounded by a thin fibro-connective wall, containing blastospores and pseudohyphae, were detected in the sub mucosa and sometimes in the muscularis mucosa. Some of these vesicles opened towards the mucosal epithelium. Fungi were Grocott (Figure 2) and PAS positive. Neither further tissue changes nor pathogens were detected in the remaining organs. The PCR revealed an amplified product of the expected size. The sequence was analyzed and assembled by Sequencing Analysis Software (Applied Biosystem) which collect the registered data at the genetic analyzer (GenBank, NCBI, Bethesda, Maryland, 20894, USA) for the alligneament by Wu Blast 2 software (NCBI, Bethesda, Maryland, 20894, USA). The comparison with all the published sequences on GeneBank, demonstrated a major alligneament, with a 98% identify and minus penalty score for *Leucosporidium scottii* internal transcribed spacer (ITS), corresponding to the accession number GenBank: AF444496 [16]. The tests were repeated two times starting from two different DNA extraction to confirm the results and twice the obtained sequence was identified as belonging to *L. scottii* with a 100%-98% identity level. There are other very closely related sequences such as *L. creatinovora* with a 97% sequence similarity. To our sequence was assigned the accession number JX014242 indicating a 596 bp long *L. scottii* ITS region.

## Conclusions

*Leucosporidium scottii*[17] synonymous *Azymocandida scottii*[18], *Candida scottii*[19], *Vanrija scottii*[20], is a fungal species frequently isolated from Antarctic and Italian waters, in terrestrial soil, in algae and decomposing plant, in chilled beef and fish [3], with high adaptability at medium-low temperatures, being the fungus a relative mesophyle [21]. To date, in literature there are no cases of candidiasis referable to *L. scottii* in birds. Anyway different candida species have been reported in birds as commensal yeasts [4] and as causative agents of disease [5-11]. In the last ten years, the application of PCR and the creation of specific data banks for fungi, has allowed to identify and to distinguish fungi morphologically almost similar [15]. Nevertheless, in our opinion, the adverse climatic conditions found during the winter 2010 season in Sicily, and particularly in the district of Messina, with downpour and low temperatures, the lack of gas heater (often recommended in partridge farming systems), the bedding based on mixed straw infrequently replaced, favoured the development of such opportunistic fungi. Finally, the presence of fungal elements in blood and several organs, the failure of the antibiotic therapy administered, as well as typization by PCR of the fungal species found as unique pathogenic agent (test was performed twice on samples obtained from different tissues, lung and gizzard), confirmed the diagnosis of candidiasis, underlining the rarity of etiology by *L. scottii*.

Candidiasis in birds is often associated with stress and poor husbandry [10,11,22], as is the case reported here. Thus, it is likely that those factors were the real problem in this group of birds.

This report can be considered of general interest for avian practitioners, pathologists and mycologists because it represents the first case of candidiasis by *L. scottii* is reported in a bird.

## Authors’ contributions

GL necropsy work and preparation of manuscript; FM x-ray laboratory and editing manuscript; GR histology and histochemistry laboratory work; FB and SR molecular biology laboratory work; FM design of study and editing manuscript. All authors read and approved the final manuscript.
